# The formation of lipid droplets favors intracellular *Mycobacterium leprae* survival in SW-10, non-myelinating Schwann cells

**DOI:** 10.1371/journal.pntd.0005687

**Published:** 2017-06-21

**Authors:** Song-Hyo Jin, Sung-Kwan An, Seong-Beom Lee

**Affiliations:** 1 Institute of Hansen’s Disease, Department of Pathology, College of Medicine, The Catholic University of Korea, 222 Banpo-daero, Seocho-gu, Seoul, Republic of Korea; 2 Research Institute for Molecular-targeted Drugs, Department of Cosmetics Engineering, Konkuk University, 120 Neungdong-ro, Gwangjin-gu, Seoul, Republic of Korea; University of Tennessee, UNITED STATES

## Abstract

Leprosy is a chronic infectious disease that is caused by the obligate intracellular pathogen *Mycobacterium leprae* (*M*.*leprae*), which is the leading cause of all non-traumatic peripheral neuropathies worldwide. Although both myelinating and non-myelinating Schwann cells are infected by *M*.*leprae* in patients with lepromatous leprosy, *M*.*leprae* preferentially invades the non-myelinating Schwann cells. However, the effect of *M*.*leprae* infection on non-myelinating Schwann cells has not been elucidated. Lipid droplets (LDs) are found in *M*.*leprae*-infected Schwann cells in the nerve biopsies of lepromatous leprosy patients. *M*.*leprae*-induced LD formation favors intracellular *M*.*leprae* survival in primary Schwann cells and in a myelinating Schwann cell line referred to as ST88-14. In the current study, we initially characterized SW-10 cells and investigated the effects of LDs on *M*.*leprae*-infected SW-10 cells, which are non-myelinating Schwann cells. SW-10 cells express S100, a marker for cells from the neural crest, and NGFR p75, a marker for immature or non-myelinating Schwann cells. SW-10 cells, however, do not express myelin basic protein (MBP), a marker for myelinating Schwann cells, and myelin protein zero (MPZ), a marker for precursor, immature, or myelinating Schwann cells, all of which suggests that SW-10 cells are non-myelinating Schwann cells. In addition, SW-10 cells have phagocytic activity and can be infected with *M*. *leprae*. Infection with *M*. *leprae* induces the formation of LDs. Furthermore, inhibiting the formation of *M*. *leprae*-induced LD enhances the maturation of phagosomes containing live *M*.*leprae* and decreases the ATP content in the *M*. *leprae* found in SW-10 cells. These facts suggest that LD formation by *M*. *leprae* favors intracellular *M*. *leprae* survival in SW-10 cells, which leads to the logical conclusion that *M*.*leprae*-infected SW-10 cells can be a new model for investigating the interaction of *M*.*leprae* with non-myelinating Schwann cells.

## Author summary

Leprosy is a chronic infectious disease that is caused by the obligate intracellular pathogen *Mycobacterium leprae* (*M*.*leprae*). Leprosy is the leading cause of all non-traumatic peripheral neuropathies worldwide. Both myelinating and non-myelinating Schwann cells are infected by *M*.*leprae* in lepromatous leprosy, but the non-myelinating Schwann cells show greater susceptibility to *M*.*leprae* invasion. However, the effect of *M*.*leprae* infection on non-myelinating Schwann cells has not been elucidated. Our results show that SW-10 cells are non-myelinating Schwann cells. Infection with *M*. *leprae* induces lipid droplet (LD) formation. Furthermore, inhibition of *M*. *leprae*-induced LD formation enhances the maturation of phagosomes containing live *M*.*leprae* and decreases the ATP content of *M*. *leprae* in SW-10 cells, suggesting that LD formation by *M*. *leprae* favors *M*. *leprae* survival in SW-10 cells. Based on these findings, it should be clear that *M*.*leprae*-infected SW-10 cells can serve as a new model for investigating the interaction of *M*.*leprae* with non-myelinating Schwann cells.

## Introduction

Leprosy is a chronic infectious disease that is caused by the obligate intracellular pathogen *Mycobacterium leprae* (*M*.*leprae*). Although the introduction of multidrug therapy (MDT) to leprosy program in 1982 resulted in a significant reduction in the prevalence of the disease, 210,758 new leprosy cases were detected globally in 2014 [1].

Leprosy is the leading cause of all non-traumatic peripheral neuropathies worldwide. *M*.*leprae* almost exclusively infects macrophages and Schwann cells. The Schwann cells, the principal glial cells of the peripheral nervous system, provide support and nutrition to the axons of neurons and are a major target of *M*.*leprae*. Physical contact of *M*.*leprae* to Schwann cells and immune reactions against either *M*.*leprae* or the infected cells damage the peripheral nerves, which results in a demyelination of the peripheral nerve fibers, and leads to irreversible nerve damage [2–5].

Depending on the level of cellular immune response, infection with *M*.*leprae* shows a diverse clinical spectrum. At one end of the spectrum, tuberculoid leprosy, a paucibacillary type, is characterized by a well-formed granuloma and a strong T-cell immune response to *M*.*leprae*. At the opposite end of the spectrum, lepromatous leprosy, a multibacillary type, is characterized by extensive bacterial multiplication within host cells and a low cell-mediated immune response to *M*.*leprae* [6, 7].

Foamy or lipid-laden macrophages are also a hallmark of lepromatous leprosy and are referred to as Virchow or Lepra cells [8]. The lipids, which accumulate in *M*.*leprae*-infected macrophages in lepromatous leprosy lesions, are composed of host-derived oxidized phospholipids, fatty acids, and cholesterol [9, 10], and are organized in cytoplasmic organelles known as lipid droplets (LDs) that are not bound to a membrane [11]. The LDs are also found in *M*.*leprae*-infected Schwann cells in nerve biopsies from lepromatous leprosy patients [12, 13]. In addition, Mattos et al. [12, 13] reported that inhibition of *M*.*leprae*-induced LD formation decreased the viability of *M*.*leprae* in primary Schwann cells, suggesting that *M*.*leprae*-induced LD formation favors intracellular *M*.*leprae* survival in Schwann cells. However, the authors did not define whether the primary Schwann cells used in their studies were myelinating or non-myelinating.

There are two types of Schwann cells: myelinating and non-myelinating cells. Myelinating Schwann cells wrap around the axons of motor and sensory neurons to form a myelin sheath. Non-myelinating Schwann cells each surround several small diameter axons, ensheathing each in a pocket of cytoplasm. Although demyelination is the ultimate consequence of leprosy neuritis, non-myelinated fibers are also injured in leprosy [14]. *M*.*leprae* infects both myelinating and non-myelinating Schwann cells in patients with lepromatous leprosy [15, 16]. In addition, Rambukkana et al. [4] have reported that, compared with myelinating Schwann cells, the non-myelinating Schwann cell is more susceptible to *M*.*leprae* invasion and preferentially harbor *M*.*leprae*, which suggests that non-myelinating Schwann cells are a natural shelter for the multiplication of *M*.*leprae*. The effect of *M*.*leprae* infection on non-myelinating Schwann cells, however, has never been elucidated in an *in vitro* infection model. Previous studies that investigated *M*.*leprae*–Schwann cell interactions have been performed mainly in primary Schwann cells or in myelinating Schwann cell lines, such as with ST88-14 cells. Thus, we needed a non-myelinating Schwann cell line as an *in vitro* model for investigating the interaction of *M*.*leprae* with Schwann cells, since it is difficult to get enough primary non-myelinating Schwann cells from peripheral nerves to perform the experiments. We found that SW-10 cells, mouse immortalized Schwann cells, express S100, a marker for cells from the neural crest, but neither myelin basic protein (MBP), a marker for myelinating Schwann cells, nor myelin protein zero (MPZ), a marker for precursor, immature, or myelinating Schwann cells [17]. Thus, we thought that *M*.*leprae*-infected SW-10 cells could be used as a new model to investigate the interactions of *M*.*leprae* with non-myelinating Schwann cells.

In the current study, we investigated the effects of LDs on *M*.*leprae*-infected non-myelinating Schwann cells. We initially characterized SW-10 cells by examining their expression of molecules, which is classically associated with myelin and Schwann cells. We then assessed the effects of LD formation by *M*.*leprae* on the maturation of phagosomes containing *M*.*leprae* and on *M*.*leprae* survival in non-myelinating Schwann cells.

## Materials and methods

### Ethics statement

All experimental procedures were examined and approved by the Animal Research Ethics Committee of the Catholic University of Korea (CUMC-2016-0058-02), in conformity with the National Institutes of Health Guidelines.

### Reagents and antibodies

C75, Celecoxib, Hoechst 333342, Staurosporine and Auramine O were obtained from Sigma-Aldrich Co. Ltd. (St. Louis, MO). Latex beads were obtained from Polysciences (Warrington, PA). C75 and Celecoxib were dissolved in DMSO. Antibodies against S100, myelin basic protein (MBP), and myelin protein zero (MPZ) were obtained from Abcam (Cambridge, MA). Antibodies against nerve growth factor receptor (NGFR) p75, adipose differentiation-related protein (ADRP), active caspase-3, and β-actin were obtained from Millipore (Billerica, MA), Fitzgerald (Acton, MA), Cell Signaling (Danvers, MA), and Santa Cruz Biotechnology (Santa Cruz, CA), respectively. Cy3-conjugated secondary antibody, Cy5-conjugated secondary antibody, and horseradish peroxidase-conjugated secondary antibody were obtained from Jackson ImmunoResearch (West Grove, PA).

### *Mycobacterium leprae* (*Thai 53*) isolation

BALB/c nude mice were obtained from Orient Bio (Seong Nam, Gyunggi-do, Korea) and maintained under specific pathogen-free conditions at the Department of Laboratory Animals, the Catholic University of Korea. Standard mouse chow (Ralston Purina, St Louis, MO) and water were provided ad libitum. The foot-pads of *M*. *leprae*-infected BALB/c *nude* mice were treated with potadine solution and washed with ice-cold DPBS to remove exogenous contamination. The foot-pads were excised, cut into small pieces, and ground with a MACs isolator (Miltenyl Biotec, Teterow, Germany). The extract was filtered using a cell strainer (BD Falcon, Durham, NC) to remove tissue debris and centrifuged at 3,000 rpm (Rotanta 460R, Hettich, Japan) for 25 min at 4°C. The pellet was resuspended in 1 ml of ice-cold DPBS and treated with 2 N sodium hydroxide for 5 min. Adding 13 ml of ice-cold DPBS neutralized the reaction. After centrifugation and resuspension, acid-fast staining was performed and the number of bacteria was counted under an oil immersion field of light microscopy using a procedure established by Shepard and McRae [18].

### Cell culture

The SW-10 (CRL-2766), a mouse neuronal Schwann cell line, was acquired from ATCC (Manassas, VA) and grown as described previously [17]. The cells were cultured in DMEM (Biowest, Lane Riverside, MO) supplemented with 10% fetal bovine serum (Biowest) and antibiotics (Gibco, Grand Island, NY).

### Immunocytochemistry

For immunostaining, the cells were fixed in 4% paraformaldehyde in PBS. The fixed cells were rinsed with PBS and incubated in blocking solution (5% goat serum and 0.001% Tween-20 in TBS) for 20 min. The cells were then incubated overnight with antibody against NGFR p75, S100, MBP, MPZ or active caspase-3 in an incubation solution (5% goat serum and 0.1% Tween-20 in TBS) at 4°C. After washing with PBS, the cells were incubated with a rabbit Cy5- or a rat Cy5-conjugated secondary antibody at room temperature for 2 h. Nuclei were counterstained for 15 min with 10 μM Hoechst 33342 (Sigma-Aldrich Co. Ltd). The negative control was processed without the presence of the primary antibody. Immunofluorescence was visualized by confocal microscope (LSM 500 Meta, Zeiss, Germany).

### Phagocytosis assay

The SW-10 cells were plated in 96-well plates and pretreated with Cytochalasin D, an inhibitor of actin polymerization, at the designated concentration for 1 h. The cells were incubated with pre-labeled Zymosan (1x10^6^ particles) for 2 h. The amount of engulfed Zymosan particles was determined using the CytoSelect 96-well phagocytosis Zymosan Colorimetric assay (Cell Biolabs, SanDiego, CA). The absorbance was measured by 405 nm in a μQuant Universal Microplate Spectrophotometer (Bio-Tek, Winooski, VT).

### Infection of *M*. *leprae*

The SW-10 cells were cultured on coverslide in a 6-well plate. The cells were infected with *M*. *leprae* at multiplicities of infection (MOI) of 10:1, 20:1, 50:1 and 100:1 for 6 h at 37°C. For complement opsonization, appropriate concentrations of *M*. *leprae* were suspended in SW-10 cells culture media containing 10% human serum (Sigma-Aldrich Co. Ltd.) as a source of complement components and incubated for 2 h at 37°C before infection. After extracellular *M*. *leprae* were washed with PBS, *M*. *leprae* were stained with AFB or Auramine O, and examined in the oil immersion field of a light microscope.

### Phagosome maturation assay

The SW-10 cells were cultured in 4-channel chamber slides (Lab-Tek II chamber slide, Thermo Fisher Scientific, Waltham, MA) and incubated overnight at 37°C under 5% CO_2_. The cells were pretreated with 20 μM Celecoxib or 157 μM C-75 for 1 h. The cells were infected with *M*. *leprae* (MOI 100:1) for 6 h and washed with warmed complete media to remove extracellular bacteria. Pre-warmed complete media was added to the cells, followed by the incubation for another 24 h. During the final 2 h of incubation, the cells were incubated with medium containing 250 nM LysoTracker Red DND-99 (Molecular Probes, Eugene, OR) according to the manufacturer’s instructions. The cells were fixed in 2% paraformaldehyde for 30 min. The level of co-localization of Auramine O-labeled *M*. *leprae* and LysoTracker was analyzed using the ZEN program (Zeiss, Oberkochen, Germany) under a LSM 510 Meta confocal microscope (Zeiss, Oberkochen, Germany).

### Transmission electron microscopy

The SW-10 cells were fixed with 2.5% glutaraldehyde for 2 h. The cells were then post fixed by treatment with 1% osmium tetroxide, dehydrated in ethanol, and embedded in Epon 812 (Polyscience, Warrington, PA). Ultrathin sections were contrasted with uranyl acetate and lead citrate. The Sections were examined via transmission electron microscopy (JEOL, Arishima, Japan).

### Western blotting analysis

At designated times, the treated cells were removed from the incubator and placed on ice. The cells were then washed 3 times with ice-cold PBS and lysed for 30 min with RIPA lysis buffer [50 mM Tris–HCl (pH 7.4), 1% Triton X-100, 150 mM NaCl, 0.1% SDS, 0.5% sodium deoxycholate, 100 mM phenylmethylsulfonyl fluoride, 1 μg/ml of leupeptin, 1 mM Na3VO4, and 1× Complete Protease Inhibitor Cocktail (Santa Cruz Biotechnology)]. Equal amounts of protein were loaded onto 10–15% SDS-PAGE gels, electrophoresed, and transferred onto PVDF membranes (Millipore, Bedford, MA). The membranes were blocked in Tris-buffered saline with 0.05% Tween 20 (TBST) supplemented with 5% powdered milk, and then incubated with primary antibody against ADRP or β-actin. The blots were then washed with TBST and incubated with a horseradish peroxidase-conjugated secondary antibody in TBST plus a 5% solution of powdered milk. The bound antibodies were detected with Amersham ECL Prime Western Blotting Detection (GE Healthcare, Buckingharmshire, UK).

### Inhibitor treatment

The SW-10 cells were plated on 6-well plates. After 24 h, the cells were pre-treated with the designated inhibitors for 1 h, followed by incubation with *M*. *leprae* at the indicated MOI. After incubation for the designated times, the cells were harvested for the next experiment. The concentrations of inhibitors used were as follows: Celecoxib, 20 μM and C-75, 157 μM. None of the inhibitors used had a significant effect on the viability of SW-10 cells.

### *M*. *leprae* ATP assay

The *M*. *leprae-*infected SW-10 cells were lysed with 0.1N NaOH for 5 min at room temperature and centrifuged 10,000 g for 5 min. The bacilli were washed three times with PBS. ATP was extracted from bacilli by the modified Tris-boiling method [19]. The bacilli were suspended in 50 μl the lysis reagent [sodium dodecyl sulfate (2%), triton X-100 (10%) and Tris-EDTA buffer (pH 8.0)], then heated at 100°C for 5 min, and cooled on ice for 1 min. The suspension was diluted with 250 μl of deionized water. The amount of ATP was quantified using the BacTiter-Glo Microbial Cell Viability Assay kit (Promega, Madison, WI), according to the manufacturer’s instructions. Briefly, 100 μl of each diluted sample was mixed with an equal volume of freshly prepared BacTiter-Glo reagent in black 96-well plate and incubated for 5 min in the dark. The emitted luminescence was detected using a SpectraMax L microplate reader (Molecular Device, Sunnyvale, CA).

### Statistical analysis

All results are expressed as the means ±SD. of data from at least three separate experiments. Statistical significance was determined via the Student’s t-test for two points or one-way ANOVA. p<*0*.*05* was considered to be statistically significant.

## Results

### SW-10 cells express S100 and NGFR p75, but neither MBP nor MPZ

**Fig 1:**
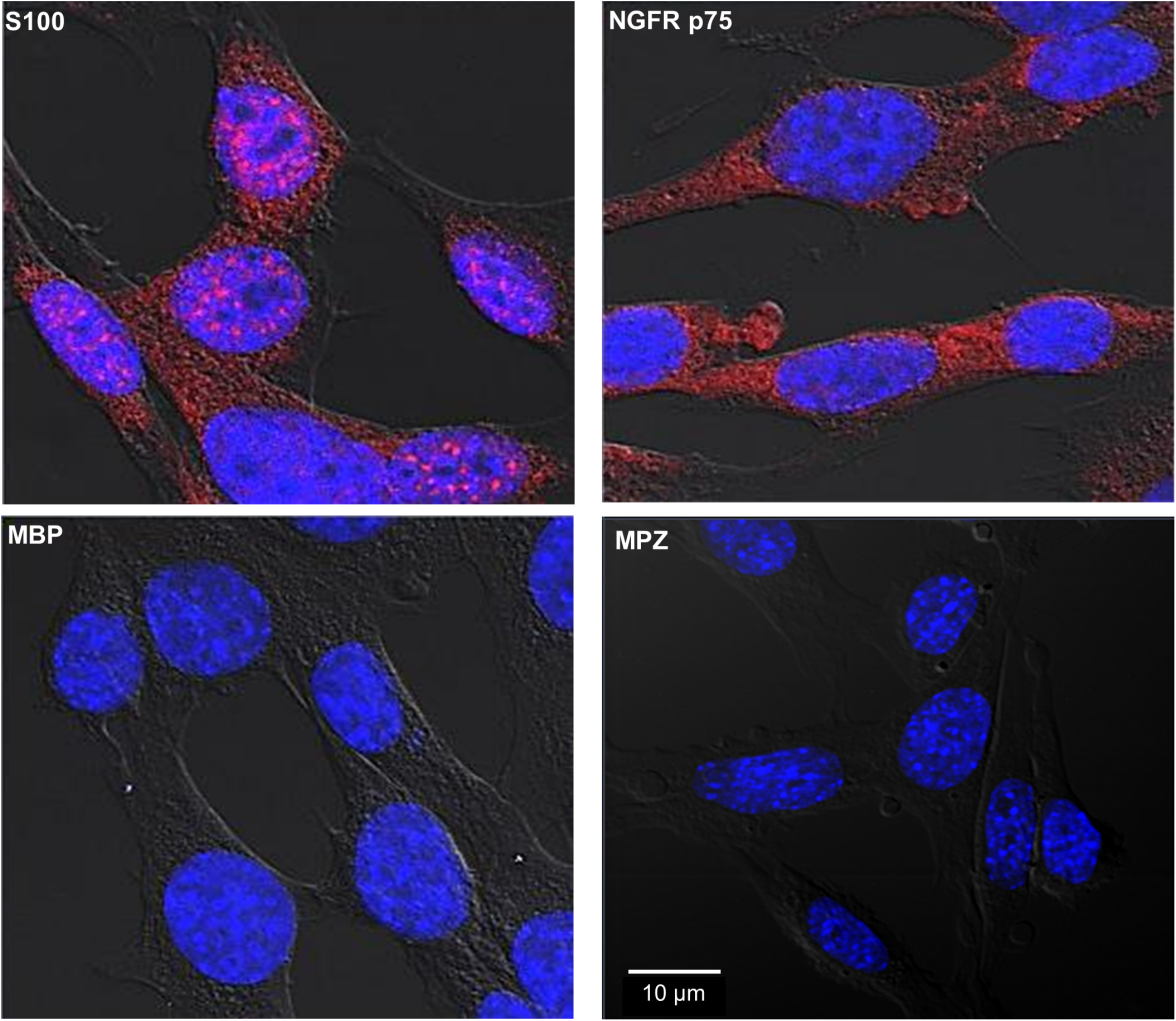
SW-10 cells express S100 and NGFR p75, but neither MBP nor MPZ. SW-10 cells were immunostained with antibodies against S100, NGFR p75, MBP, or MPZ. Nuclei were counterstained for 15 min with 10 μM Hoechst 33342 (Sigma-Aldrich Co. Ltd). Scale bar: 10 μm.

We initially characterized SW-10 cells, a mouse Schwann cell line. As shown in Fig 1, SW-10 cells express S100, a marker for cells from neural crest, and NGFR p75, a marker for immature or non-myelinating Schwann cell, but neither MBP, a marker of myelinating Schwann cell, nor MPZ, a marker for precursor, immature, or myelinating Schwann cells [17, 20]. Thus, these results indicate that SW-10 cells are non-myelinating Schwann cells.

### SW-10 cells have phagocytic activity

**Fig 2:**
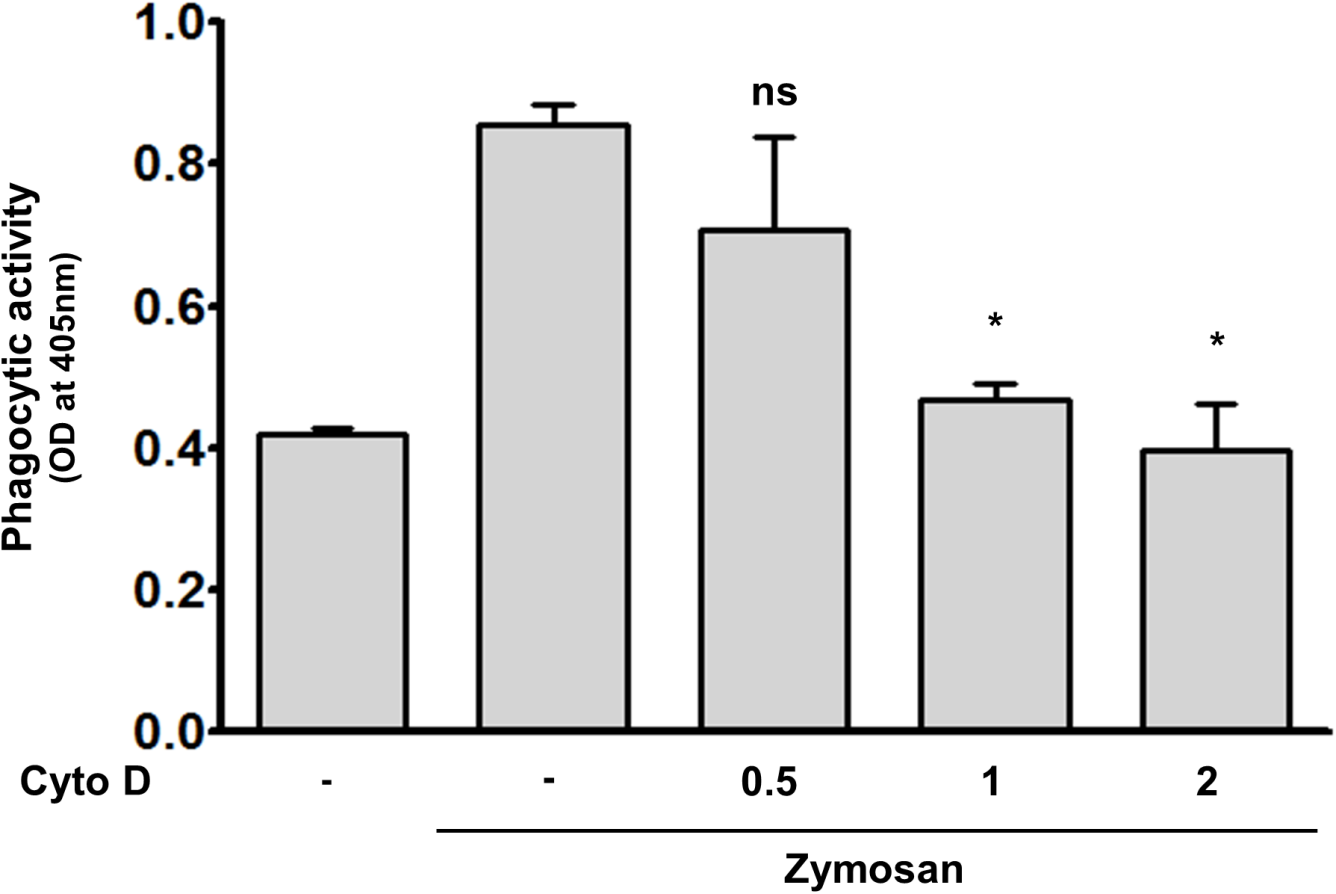
SW-10 cells have phagocytic activity. The phagocytic activity of SW-10 cells was determined using a CytoSelect 96-well phagocytosis assay kit (Cell BioLabs). Cells were pre-treated with cytochalasin D, an inhibitor of phagocytosis and actin polymerization, at the designated concentration for 1h at 37°C, and then incubated with Zymosan particles at a 100:1 ratio for 2 h. Significance was calculated via one-way ANOVA. *P <0.05 versus control cells with only Zymosan uptake. Cyto D: cytochalasin D.

Schwann cells are well known to have phagocytic activity. We used the CytoSelect 96-well phagocytosis Zymosan colorimetric assay (Cell Biolabs) to investigate the possibility that SW-10 cells could have phagocytic activity. As shown in Fig 2, SW-10 cells phagocytosed Zymosan. In addition, pre-treatment with Cytochalasin D, an inhibitor of phagocytosis and actin polymerization, inhibited the phagocytic activity of Schwann cells (Fig 2). These results indicate that SW-10 cells have phagocytic activity.

### *M*.*leprae* infected SW-10 cells

**Fig 3:**
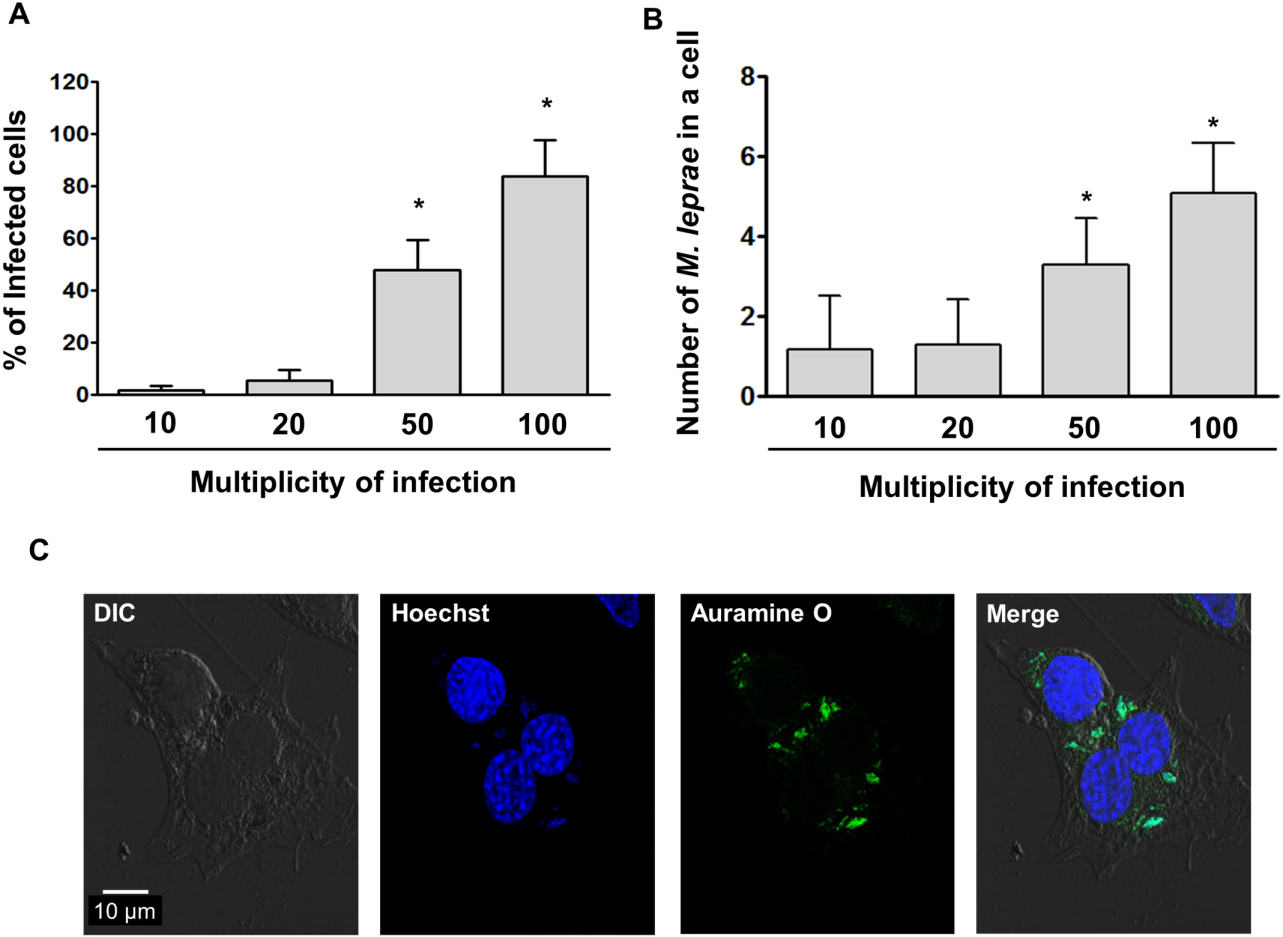
SW-10 cells are infected with *M*. *leprae*. (A and B) SW-10 cells were incubated with *M*. *leprae* at the MOI of 10:1, 20:1, 50:1 and 100:1 for 6 h at 37°C. After extracellular *M*. *leprae* were washed away, *M*. *leprae* were stained with Auromine O. The percentage of *M*. *leprae*-infected cells and the number of *M*. *leprae* in a cell were determined in the oil immersion field of a light microscope. Significance was calculated via one-way ANOVA. *P <0.05 versus cells were incubated with *M*. *leprae* at the MOI of 100:1. (C) SW-10 cells were incubated with *M*. *leprae* at the MOI of 100:1 for 6 h at 37°C. After extracellular *M*. *leprae* were washed away, *M*. *leprae* were stained with Auramine O. Nuclei were counterstained for 15 min with 10 μM Hoechst 33342 (Sigma-Aldrich Co. Ltd). Scale bar: 10μm.

*M*.*leprae* almost exclusively infects macrophages and Schwann cells. We investigated whether *M*.*leprae* infects SW-10 cells. SW-10 cells were incubated with *M*. *leprae* using MOI of 10:1, 20:1, 50:1 and 100:1 for 6 h at 37°C. At the MOI of 100:1, 83.6% of cells were infected with *M*.*leprae* and the average number of *M*.*leprae* in a cell was 5.1 (Fig 3). Based on these findings, for the remainder of the current study we incubated SW-10 cells with *M*. *leprae* at the MOI of 100:1.

### *M*.*leprae* infection does not induce apoptosis in SW-10 cells

**Fig 4:**
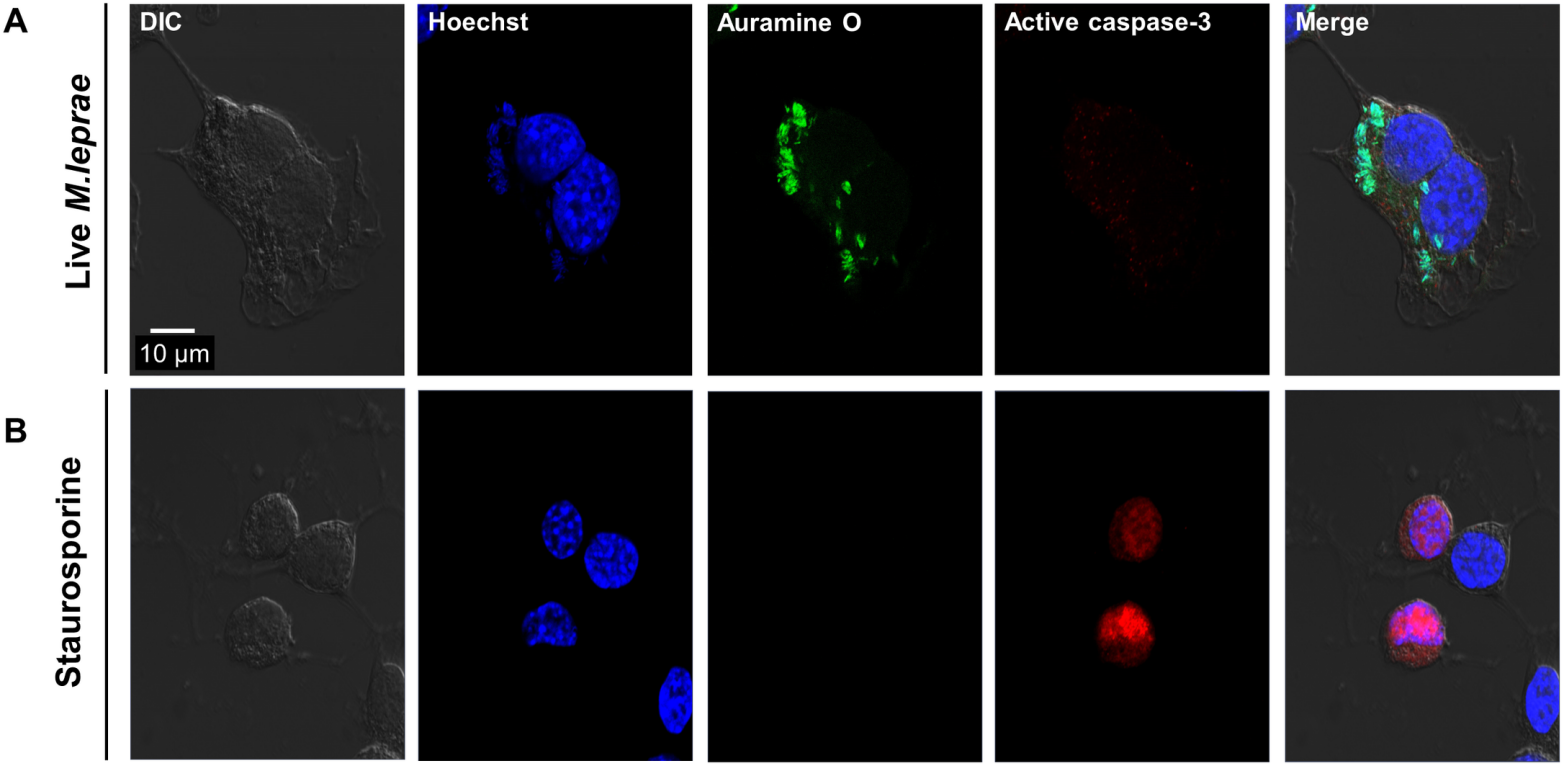
*M*.*leprae* infection does not induce apoptosis in SW-10 cells. (A) SW-10 cells were incubated with *M*. *leprae* at the MOI of 100:1 for 6 h at 37°C. After extracellular *M*. *leprae* were washed away, the cells were again incubated for another 48 h. *M*. *leprae* were stained with Auramine O and the cells were immunostained with antibodies against active caspase-3. (B) SW-10 cells were treated with 1 μM staurosporine for 2 h. The cells were then immunostained with antibodies against active caspase-3. Nuclei were counterstained for 15 min with 10 μM Hoechst 33342 (Sigma-Aldrich Co. Ltd). Scale bar: 10μm.

Apoptosis of Schwann cells is frequently found in human leprosy lesions [21]. Thus, we investigated whether *M*.*leprae* infection induces apoptosis in SW-10 cells. However, under our experimental conditions, *M*.*leprae* infection did not induce the expression of active caspase-3, an indicator of apoptosis, in SW-10 cells, whereas treatment with 1 μΜ staurosporine, a well-known inducer of apoptosis, induced the expression of active caspase-3 (Fig 4).

### *M*. *leprae* induces lipid droplet formation in SW-10 cells

**Fig 5:**
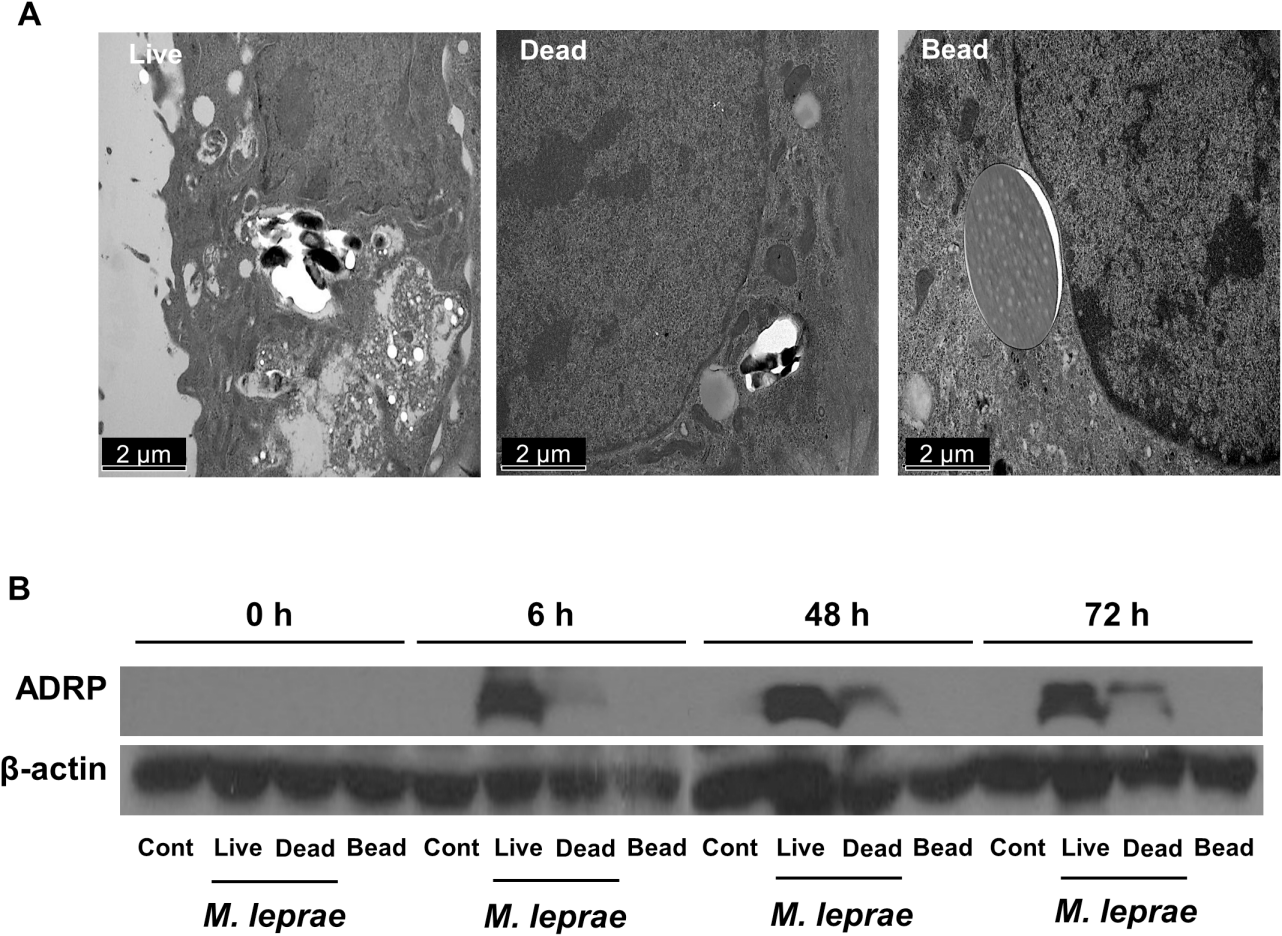
Live *M*. *leprae* induces the formation of LDs in SW-10 cells. SW-10 cells were incubated with either live or dead *M*.*leprae*, or latex beads (3.0 μm and Polysciences) at a MOI of 100:1 for 6 h at 37°C. After the extracellular *M*. *leprae* were washed away, the cells were again incubated either for another 48 h (A) or for the indicated times (B). The formation of LDs was examined by transmission electron microscopy (A). The expression of ADRP was determined by western blot analysis (B). Similar results were observed in three independent experiments. Scale bar: 2 μm.

*M*. *leprae* infection is known to induce the formation of LDs in primary Schwann cells and in ST 88–14 cells, which make up the human Schwann cell line [12, 13]. We investigated whether *in vitro M*. *leprae* infection induces the formation of LDs in SW-10 cells. As shown in Fig 5A, infection with live *M*. *leprae*, but neither dead *M*. *leprae* nor latex bead, induced the formation of LDs in SW-10 cells. Consistent with these results, live *M*. *leprae* induced the expression of ADRP, a marker of LD, whereas dead *M*. *leprae* or latex bead did not affect expression (Fig 5B).

### Treatment with celecoxib or C-75 reverses the fusion impairment of *M*.*leprae*-containing phagosome with lysosome in SW-10 cells

Treatment with NS-398, a non-steroidal anti-inflammatory drug, or C-75, an inhibitor of fatty acid synthetase, inhibits *M*.*leprae*-induced prostaglandin E_2_ (PGE_2_) production and LDs formation, subsequently leading to a decrease in *M*. *leprae*-survival in primary Schwann cells [13].

**Fig 6:**
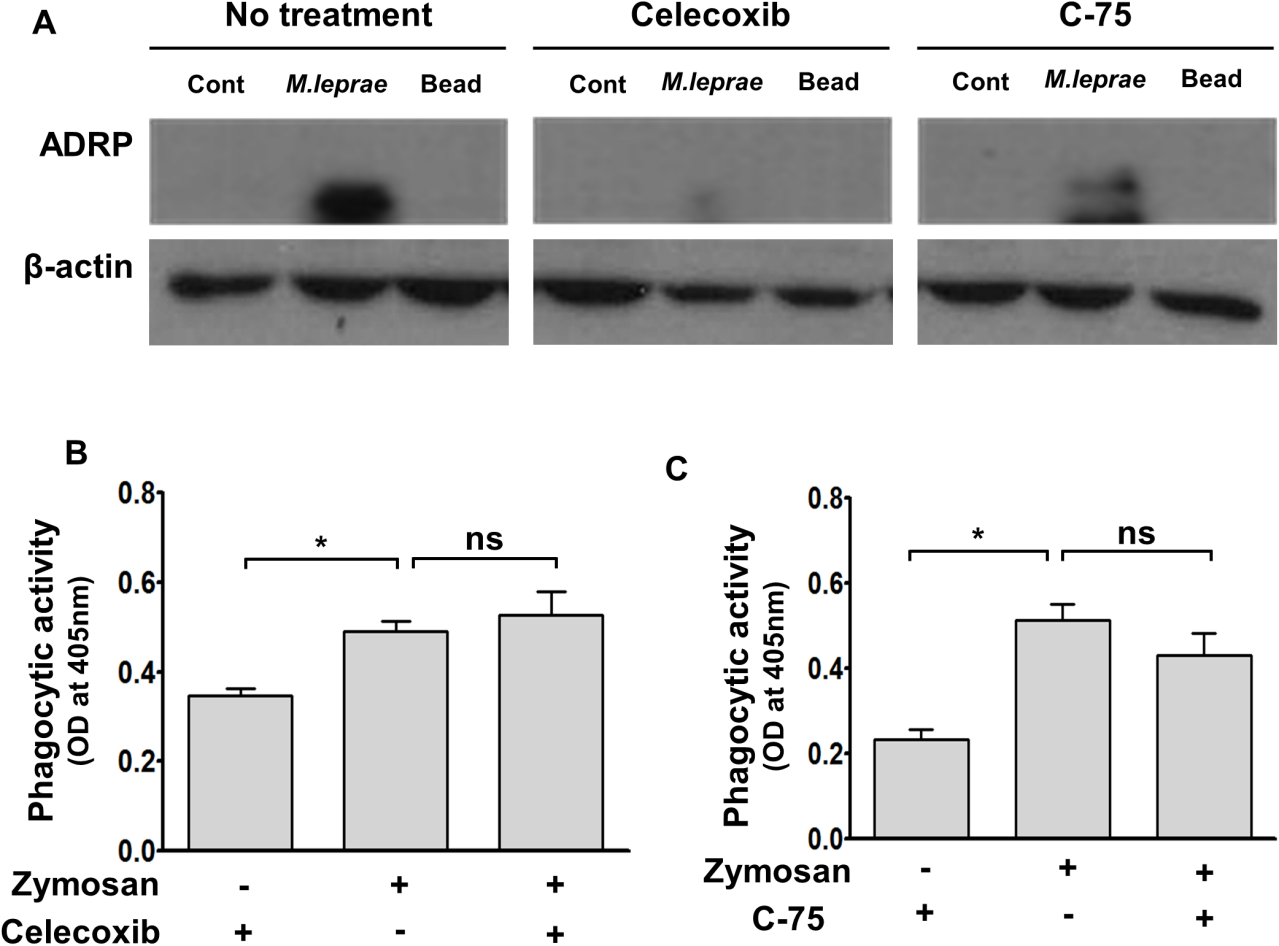
Treatment with celecoxib or C-75 inhibits *M*.*leprae*-induced ADRP expression in SW-10 cells. (A) Cells were pre-treated either with 20 μΜ celecoxib, an inhibitor of COX-2, or with 157 μΜ C-75, an inhibitor of FAS, for 1 h, and were then incubated with live *M*. *leprae* or latex beads (3.0 μm, Polysciences) at a MOI of 100:1 for 6 h at 37°C. The expression of ADRP was determined by western blot analysis. (B and C) The effect of celecoxib and C-75 on the phagocytic activity of SW-10 cells was determined using the CytoSelect 96-well phagocytosis assay kit (Cell BioLabs). Cells were pre-treated with celecoxib or C-75, and were then incubated with Zymosan particles at a 100:1 ratio for 2 h. Significance was calculated via one-way ANOVA. *P <0.*05* versus control cells with only Zymosan uptake.

**Fig 7:**
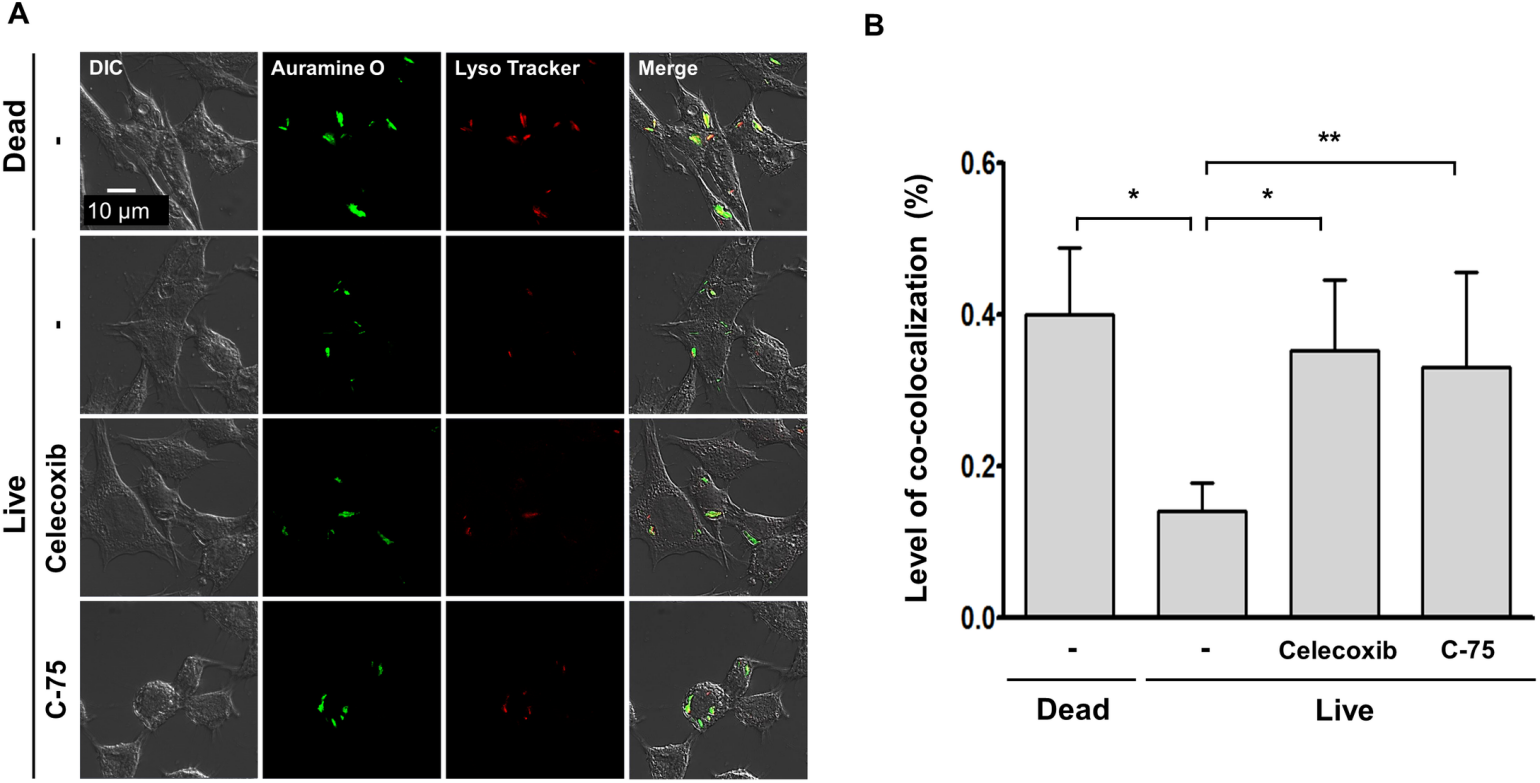
Treatment with celecoxib or C-75 reverses impairment of the fusion of live *M*.*leprae*-containing phagosomes with lysosomes in SW-10 cells. (A and B) Cells were pre-treated either with 20 μΜ celecoxib, an inhibitor of COX-2, or with 157 μΜ C-75, an inhibitor of FAS, for 1 h, and were then incubated with either live or dead *M*. *leprae* at a MOI of 100:1 for 6 h at 37°C. After the extracellular *M*. *leprae* were washed away, the cells were again incubated for another 48 h and then were stained with Auramine O and a red-fluorescent dye for labeling and tracking acidic organelles, LysoTracker Red (Molecular probes). The maturation level of the phagosomes containing live *M*. *leprae* was determined by measuring the co-localization level of Auramine O-labeled *M*.*leprae* with Lyso Tracker Red (Molecular probes). Nuclei were counterstained for 15 min with 10 μM Hoechst 33342 (Sigma-Aldrich Co. Ltd). *P <0.*05* and **P <0.*01* between the indicated groups (B). Scale bar: 10 μm.

**Fig 8:**
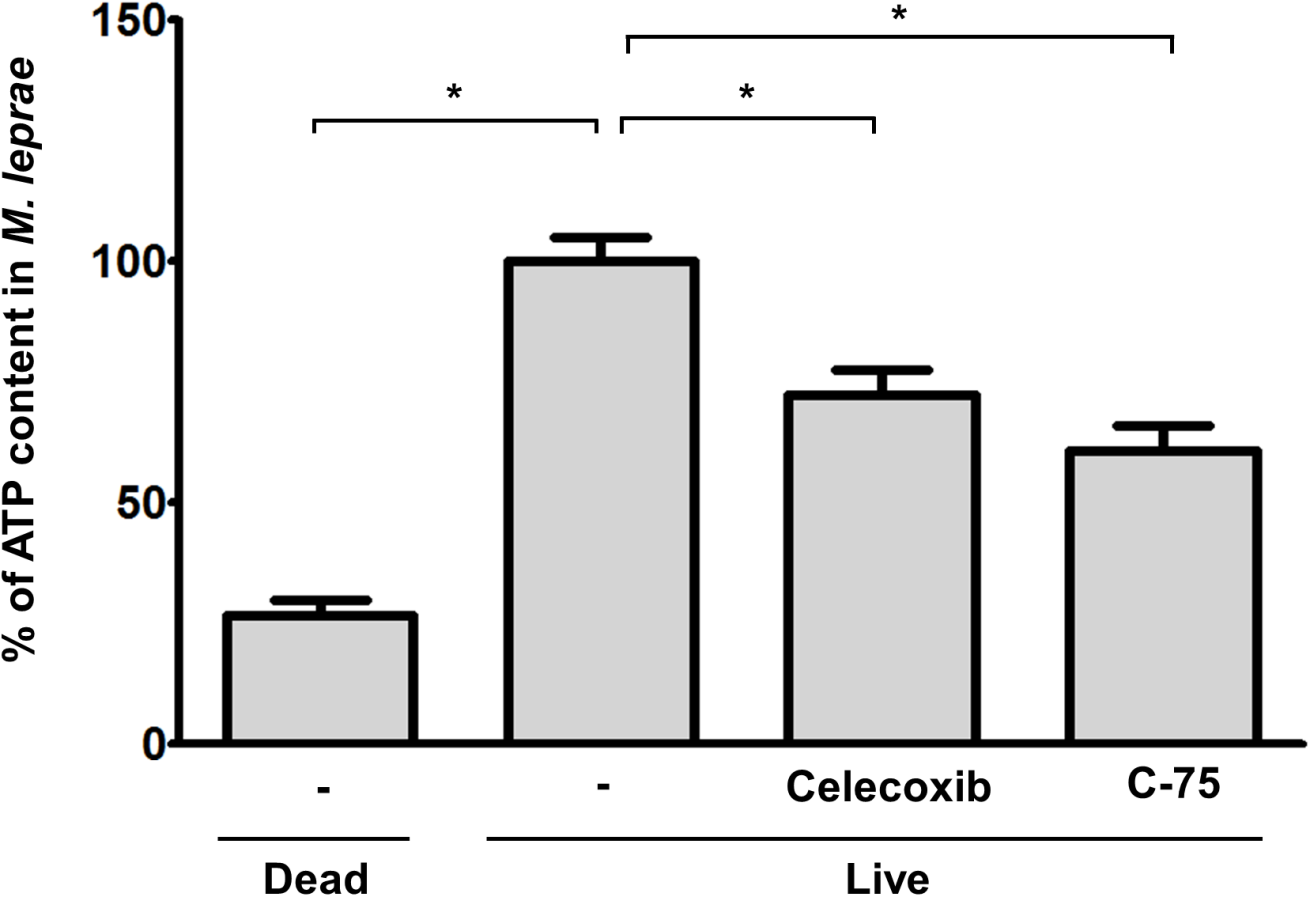
Treatment with celecoxib or C-75 reduces the ATP content of *M*.*leprae* in SW-10 cells. Cells were pre-treated with 20 μΜ celecoxib, an inhibitor of COX-2, or 157 μΜ C-75, an inhibitor of FAS, for 1 h, and then incubated with live *M*. *leprae* at the MOI of 100:1 for 6 h at 37°C. After extracellular *M*. *leprae* were washed away, the cells were again incubated for another 72 h. The *M*. *leprae-*infected SW-10 cells were lysed with 0.1 N NaOH for 5 min. The amount of ATP was quantified using the BacTiter-Glo Microbial Cell Viability Assay kit (Promega), according to the manufacturer’s instructions. *P <0.*05* between the indicated groups.

Thus, we used celecoxib, an inhibitor of cyclo-oxygenase (COX)-2, or C-75, an inhibitor of fatty acid synthetase to investigate the effect that the LD formation caused by *M*.*leprae* can exert on the maturation of phagosomes containing live *M*.*leprae* and on *M*. *leprae* survival in SW-10 cells. Treatment with celecoxib or C-75 did not affect the phagocytic activity of SW-10 cells (Fig 6B and 6C), but it inhibited *M*.*leprae*–induced ADRP expression (Fig 6A), which resulted in an increase in the co-localization of Auromine O-labelled *M*.*leprae* with Lysotracker, a marker of lysosome (Fig 7A and 7B) and in a subsequent decrease in the ATP content of *M*.*leprae* in SW-10 cells (Fig 8). Taken together, our results show that LD formation by *M*.*leprae* also favors *M*.*leprae* survival in non-myelinating Schwann cells.

## Discussion

The findings reported herein indicate that SW-10 cells express S100, a marker for cells from the neural crest, and NGFR p75, a marker for immature or non-myelinating Schwann cells, but neither MBP, a marker of myelinating Schwann cells, nor MPZ, a marker for precursor, immature, or myelinating Schwann cells [17, 20], which suggests that SW-10 cells are non-myelinating Schwann cells (Fig 1). In addition, SW-10 cells have phagocytic activity (Fig 2) and are subjected to infection with *M*. *leprae* (Fig 3). Infection with *M*. *leprae* induces LD formation (Fig 5). Furthermore, the inhibition of *M*. *leprae*-induced LD formation enhances the maturation of phagosomes containing live *M*.*leprae* (Fig 7) and decreases the ATP content of *M*. *leprae* (Fig 8) in SW-10 cells, which suggests that LD formation by *M*. *leprae* favors *M*. *leprae* survival in SW-10 cells. These results support the role of non-myelinating Schwann cells as a shelter for *M*. *leprae* multiplication.

Because *M*. *leprae* preferentially infects Schwann cells of the peripheral nerves in cooler regions of the body, the temperature below the body’s core temperature (37°C) is suggested to be ideal condition for the growth and maintenance of *M*. *leprae*. In experimental conditions, an incubation of 33°C is also suggested as optimal for studying the effect of *M*. *leprae* on Schwann cells. Hagge et al. [22] reported that *M*. *leprae* showed 56% viability in Schwann cells for 3 weeks after infection at 33°C, compared with 3.6% viability at 37°C. However, Itty et al. [23] reported that the number of *M*. *leprae* adhering to mouse primary Schwann cells during incubation at 37°C was more than twice as great as that at 34°C. Since the adherence of *M*. *leprae* to Schwann cells is a prerequisite for phagocytosis and our results focused on the early (24–72 h) effects of *M*. *leprae* infection on Schwann cells, we performed *in vitro* infections of Schwann cells with *M*. *leprae* at 37°C.

Adenosine-5-triphosphate (ATP) is used in all living cells as a co-enzyme for energy transfer. The ATP content is fairly constant for each cell type and decreases quickly following cell death [19]. In the current study, we determined the viability level of *M*. *leprae* that is derived from SW-10 cells by measuring its ATP content (Fig 8). Katoch et al. [24] have reported that the ATP content of cultivable mycobacteria, *M*. *tuberculosis* and *M*. *lufu*, directly correlated with viable numbers of mycobacteria. In addition, measuring the ATP content of *M*. *leprae* has also been used as an *in vitro* method to determine the viability of *M*. *leprae* [19, 24].

LDs are cytoplasmic lipid storage organelles that are found in most eukaryotic cells. LDs composed of a hydrophobic core of neutral lipids (triglycerides and cholesterol esters) surrounded by a phospholipid monolayer and by specific proteins, including perilipin, ADRP, and tail-interacting protein 47 (TIP47) [25, 26]. LDs basically play an important role in lipid metabolism and provide a site for the generation of inflammation mediators, prostaglandins and leukotrienes [13, 27]. These LDs also support pathogen growth or survival in host cells [28]. LDs serve as a site for the replication of viruses, including the hepatitis C virus [29], Dengue virus [30], and Rotavirus [31]. LDs are also used for the survival of *Chlamydia trachomatis*, an obligate intracellular bacteria [32, 33]. In addition, LDs are also involved in the pathogenesis of mycobacterial infection. Infection with *M*.*tuberculosis* induces the formation of LDs in macrophages [34]. In lepromatous leprosy, *M*.*leprae*-infected macrophages in dermal lesions and Schwann cells in peripheral nerves show a foamy, lipid-laden appearance [8, 11]. The foamy appearance is at least in part derived from the accumulation of LDs in *M*.*leprae*-infected cells [8, 11]. LDs are recruited to *M*.*leprae*-containing phagosome in *M*.*leprae*-infected Schwann cells [12]. Although how LDs inhibit the maturation of *M*.*leprae*-containing phagosomes in Schwann cells is unknown, *M*.*leprae*-induced LD formation favors intracellular *M*.*leprae* survival in primary Schwann cells [12, 13]. Consistent with these results, our results also show that LDs formation by live *M*.*leprae* inhibits the maturation of *M*.*leprae*-containing phagosome, leading to an increase in the viability of *M*.*leprae* in SW-10 cells, non-myelinating Schwann cells (Figs 7 and 8).

*M*.*leprae* infection induces demyelination and axonal injury of peripheral nerves via the immune reaction to *M*.*leprae*-infected cells and/or via the physical contact of *M*.*leprae* to Schwann cells [2–5]. Apoptotic Schwann cells are frequently found in human leprosy lesions [21]. *M*.*leprae* infection induces apoptosis in ST88-14 cells, a human Schwann cell line [35]. In addition, treatment with *M*.*leprae* 19-kDa lipoprotein induces apoptosis in ST88-14 and in primary human Schwann cells [21]. However, our results show that *in vitro* infection with *M*.*leprae* does not induce apoptosis in SW-10 cells (Fig 4). Consistent with our results, Rambukkana et al. [3] reported that in *in vitro* and *in vivo* infection models, non-myelinating Schwann cells harbor *M*.*leprae* in large numbers rather than showing apoptosis. Thus, further detailed studies are needed to assess the influence of *M*.*leprae* on myelinating and non-myelinating Schwann cells through a pathway that is either dependent or independent of immune reactions.

Upon nerve damage, *M*.*leprae* invades Schwann cells where it can survive for long periods [36]. In addition, *M*.*leprae* infection reprograms the adult Schwann cells to a stem cell type, which promotes the dissemination of *M*.*leprae* [37]. Although *M*.*leprae* infection is detected in both myelinating and non-myelinating Schwann cells of patients with lepromatous leprosy [15, 16], *M*.*leprae* preferentially invades the non-myelinating Schwann cells, where it multiplies, releases, and re-infects more non-myelinating Schwann cells [3, 4]. However, the effect of *M*.*leprae* infection on non-myelinating Schwann cells has not been elucidated. We wondered if SW-10 cells would be non-myelinating Schwann cells, which would make them targets of *M*.*leprae*. Our results indicate that SW-10 cells show similar phenotypes in response to *M*.*leprae* infection, as shown in primary Schwann cells and in ST88-14 cells, which is the myelinating Schwann cell line (Table 1) [35]. The results of this study suggest that *M*.*leprae*-infected SW-10 cells could be a new model that can be used to investigate the interactions of *M*.*leprae* with non-myelinating Schwann cells.

**Table 1:** Comparison of Schwann cell lines as *in vitro* models for investigating the interaction of *M*.*leprae* with Schwann cells.

		Primary Schwann cells [12, 13]	ST88-14 cells [13, 35]	SW-10 cells [17, current study]
**Origins**		Human or mouse, Peripheral nerves [12, 13]	Human malignant Schwannoma [13, 35]	Mouse immortalized Schwann cell line
**Cell surface marker**	S100 [20] (marker for cells from neural crest)	Positive [12, 13]	Positive [35]	Positive [17, current study]
	NGFR p75 [20] (marker for immature or non-myelinating Schwann cells)	ND	ND	Positive [current study]
	MBP [20] (marker for myelinating Schwann cells)	ND	Positive [35]	Negative [17, current study]
	MPZ [20] (marker for precursor, immature, or myelinating Schwann cells)	ND	ND	Negative [17, current study]
***M*.*leprae*-induced apoptosis**		ND	Positive [35]	Negative [current study]
***M*.*leprae*-induced LD biogenesis**		Positive [12, 13]	Positive [13]	Positive [current study]
**LD formation favors *M*.*leprae* intracellular survival in Schwann cells**		Positive [12, 13]	ND	Positive [current study]

ND, not defined; LD, lipid droplet.

NGFR, nerve growth factor receptor; MBP, myelin basic protein; MPZ, myelin protein zero
